# Supplementary material for: Communicating about Energy Policy in a Resource-Rich Jurisdiction during the Climate Crisis: Lessons from the People of Brisbane, Queensland, Australia
Source: Int J Environ Res Public Health. 2022 Apr 12;19(8):4635. doi: 10.3390/ijerph19084635 (PMC9029168; doi:10.3390/ijerph19084635)
Supplement: Supplementary file 1 [file ijerph-19-04635-s001.zip › ijerph-1650016-supplementary.pdf]

Public attitude to environmental policy.

Semi-structured interview guide

Thank you for agreeing to talk with me today. Before I begin, I would like to remind you that you don't have to answer any questions that you don't want to. If you don't want to answer a particular question, just tell me that and I will move on to the next one.

1. What environmental issues in Australia are you concerned about?

2. Who do you think should be responsible for addressing these (or this) issue(s)?

3. What do you think about the idea that 100% of Australia's electricity should be sourced from renewable energy technologies?

4. What do you think about the idea that government should provide support to coal mining towns so that they can transition to alternative industries?

5. What do you think about the idea that there should be no new coal mines in Queensland or in the rest of Australia?

6. What do you think about the idea that there should be no new gas fields opened up for gas extraction in Queensland or in the rest of Australia?

7. What do you think about the idea that the Adani coal mine should be stopped from going ahead?

8. Is there anything else that you would like to add or tell me about?

9. I would now like to ask you some short questions about yourself:

    a. Postcode where you live
    b. Age category: 18 – 24, 25 – 29, 30 – 39, 40 – 50, 50 – 64, 65 or older?
    c. Do you have any children living at home? If so, how many?
    d. What was your highest level of education attainment?
    e. What is your occupation?
    f. What political party did you vote for in the last Federal election?
